# Mouse Testis Development and Function Are Differently Regulated by Follicle-Stimulating Hormone Receptors Signaling During Fetal and Prepubertal Life

**DOI:** 10.1371/journal.pone.0053257

**Published:** 2012-12-27

**Authors:** Stéphanie Migrenne, Evelyne Moreau, Pirjo Pakarinen, Andrée Dierich, Jorge Merlet, René Habert, Chrystèle Racine

**Affiliations:** 1 University Paris Diderot, Sorbonne Paris Cité, Laboratory of Development of the Gonads, Unit of Stem Cells and Radiation, Fontenay-aux-Roses, France; 2 CEA, DSV, iRCM, SCSR, LDG, Fontenay-aux-Roses, France; 3 INSERM, Unité 967, Fontenay-aux-Roses, France; 4 University of Turku, Institute of Biomedicine, Department of Physiology, Turku, Finland; 5 CNRS 7104, IGBMC, Illkirch, France; 6 INSERM, U964, Illkirch , France; University Hospital of Münster, Germany

## Abstract

It is currently admitted that Follicle-Stimulating Hormone (FSH) is physiologically involved in the development and function of fetal/neonatal Sertoli cells in the rat but not the mouse. However, FSH is produced by both species from late fetal life onwards. We thus reinvestigated the role of FSH in mouse testis development at day 0 (birth) 6, 8 and 10 post-partum (dpp) by using mice that lack functional FSH receptors (FSH-R^−/−^). At birth, the number and proliferative index of Sertoli cells were significantly lower in FSH-R^−/−^ mice than in wild type neonates. Claudin 11 mRNA expression also was significantly reduced in FSH-R^−/−^ testes at 0 and 8 dpp, whereas the mRNA levels of other Sertoli cell markers (Transferrin and Desert hedgehog) were comparable in FSH-R^−/−^ and wild type testes. Conversely, AMH mRNA and protein levels were higher at birth, comparable at 6 dpp and then significantly lower in FSH-R^−/−^ testes at 8–10 dpp in FSH-R^−/−^ mice than in controls. Although the plasma concentration of LH and the number of Leydig cells were similar in FSH-R^−/−^ and control (wild type), testosterone concentration and P450c17 mRNA expression were significantly increased in FSH-R^−/−^ testes at birth. Conversely, at 10 dpp when adult Leydig cells appear, expression of the steroidogenic genes P450scc, P450c17 and StAR was lower in FSH-R^−/−^ testes than in controls. In conclusion, our results show that 1) like in the rat, signaling via FSH-R controls Sertoli cell development and function during late fetal life in the mouse as well; 2) paracrine factors produced by Sertoli cells are involved in the FSH-R-dependent regulation of the functions of fetal Leydig cells in late fetal life; and 3) the role of FSH-R signaling changes during the prepubertal period.

## Introduction

During fetal life, Sertoli cells are the first to differentiate in the gonad anlage. These large cells adhere one to the other and surround the germ cells (or gonocytes) to form the seminiferous cords [1]. This process occurs from 13.5 to 14.5 day post-conception (dpc) in the rat and from 11.5 to 12.5 dpc in the mouse. Then, Sertoli cells continue to proliferate until day 16 post-partum (dpp) in the rat and 17 dpp in the mouse, when the Sertoli cell population is definitively established. Finally, in adulthood, spermatogenesis is quantitatively and qualitatively dependent on the number and optimal function of Sertoli cells as they provide the structural support necessary for germ cell maturation [2], [3], [4].

After the initial differentiation of Sertoli cells has occurred, Leydig cells differentiate in the interstitial space [5], [6], [7], [8]. In the rat, fetal testis begins to produce testosterone at 15.5 dpc [9], [10] and expresses 3ß-hydroxysteroid dehydrogenase (3ß-HSD) from 14.5 dpc onwards [11]. In the mouse, testosterone production is detected from 12.5 dpc onwards [12]. In the rat and mouse, the functional activity of fetal Leydig cells decreases during late fetal life and neonatal life, although this cell type may persist in adult life, at least in the rat [13]. Another population of Leydig cells (i.e. adult Leydig cells) starts to differentiate from 15 dpp in the rat [5], whereas in the mouse they replace the fetal cells between 5 and 15 dpp [14], [15], [16]. The morphology and some physiological features of adult Leydig cells differ from those of the fetal cells [5], [6], [17].

In adult testes, Follicle Stimulating Hormone (FSH) is the major endocrine regulator of Sertoli cell function. More than 300 genes regulated by FSH have been identified by oligonucleotide microarray analysis in cultured Sertoli cells from 20 dpp rat testes [18]. Furthermore, in the adult, FSH also exerts a stimulatory action on Leydig cell function [6], [19], [20], [21], [22]. As FSH receptors (FSH-R) are expressed only by Sertoli cells and there is no membrane contact between Sertoli and Leydig cells, this suggests the existence of FSH-regulated paracrine factors that influence the functions of Leydig cells. Indeed, many factors that are produced by Sertoli cells and have a positive or negative effect on Leydig cell function have been identified [5], [23].

The ontogeny of the control by FSH on the function and development of Sertoli cells is well known in the rat. In the pituitary, transcripts of FSHß gene are first detected by *in* situ hybridization at 17.5 dpc. *FSH-R* mRNA is first detected at 14.5 dpc in Sertoli cells and these cells can respond to FSH from 15.5 dpc [24], [25]. The pioneer work by Orth clearly showed that, in rats, the proliferation of Sertoli cells is FSH-dependent during late fetal life [26]. Specifically decapitation of rat fetuses at 18.5 dpc led to a decrease in Sertoli cell proliferation at 20.5 and 21.5 dpc that could be rescued by injections of FSH. Similarly, using the fetal decapitation approach, we previously demonstrated that the functions of Sertoli cells depend on the pituitary hormones [27]. Lastly, FSH can influence the *in vitro* secretion of testosterone by rat fetal testes as early as 15.5 dpc [25].

Conversely, the ontogeny of the control by FSH of fetal and neonatal Sertoli cell development and function remains unclear in the mouse. The common alpha-glycoprotein subunit is first detected in the Rathke's pouch in 11.5 dpc mice and the FSH beta subunit in the anterior pituitary at 17.5 dpc [28]. In hypogonadal (hpg) mice, which lack circulating gonadotropins, the number of Sertoli cells is normal at 18.5 dpc but it is reduced at the day of birth [29]. However, three studies from the same laboratory using *FSH* and *FSH-R* deficient mice concluded that the development and function of Sertoli and Leydig cells are independent from FSH and FSH-R signaling during fetal and early neonatal life [20], [30], [31].

It is thus necessary to reinvestigate the role of FSH in the development and functions of fetal and neonatal testicular cells. This is all the more important as fetal and neonatal testis is one of the main targets of endocrine disruptors. To this aim, we analyzed the development and functions of Sertoli, Leydig and germ cells at the end of fetal life (birth) and at 6, 8 and 10 dpp in mice lacking functional FSH-R [32].

## Experimental Procedures

### Animals

FSH-R deficient mice [32] were maintained under controlled conditions of temperature (22°C), light (12L:12D) and humidity, with food and water provided *ad libitum*. Colonies were maintained by breeding heterozygous males with heterozygous females. Males were genotyped by PCR analysis of genomic DNA using the following oligonucleotides: 5′-ATGGCCTTGCTCCTGGTCTCCTTGCTGGCA-3′ (reverse primer), 5′-AGAAAGCGAAGGAGCAAAGC-3′ (forward primer) and 5′-TAGCCTTTGGTTAGGACAGCCCTATTTCA-3′ (forward primer). The PCR conditions (40 cycles) were 96°C for 2 min, followed by 94°C for 1 min, 55°C for 2 min, 72°C for 2 min and a final extension at 72°C for 10 min. PCR products were separated on 1% agarose gels to identify the 271 bp wild type FSH-R and the 388 bp mutant FSH-R fragment. Both fragments were present in heterozygous animals (data not shown).

Since the estimated time of ovulation was 02.00 a.m., the day following the overnight mating was counted as 0.5 day post-conception (dpc). Birth occurred spontaneously between 6 p.m. on 18.5 dpc and 10 a.m. on 19.5 dpc. Day 19.5 pc were counted as 0 dpp. The sex of the animals was determined by observation of the ano-genital distance. Animals were sacrificed at 10 a.m. by decapitation and their blood and testes were immediately collected.

In some experiments, to limit individual variability and to perform paired statistical tests, only one male from each genotype was selected from the same litter for further analyses.

The animal facility is licensed by the French Ministry of Agriculture (agreement N°B92-032-02). All animal experiments were supervised by Pr. René Habert (agreement delivered by the French Ministry of Agriculture for animal experiment N°92–191) in compliance with the NIH Guide for Care and Use of Laboratory Animals [33]. All efforts were made to minimize animal suffering.

### Steroid extraction

Testosterone was extracted from testes as previously described [34]. Briefly, testes were homogenized by sonication in 2 ml ethyl acetate. After centrifugation, the ethyl acetate phase was removed and kept. A second extraction was performed and the combined ethyl acetate phases were vortexed for 1 min, dried under a heated stream and re-dissolved at 50°C in 500 µl RIA buffer for radioimmunoassay.

### Testosterone RIA Assay

Testosterone concentration was measured by using a specific RIA assay as described [34]. Briefly, 100 µl reconstituted testicular extracts or testosterone standards were incubated at 4°C with 100 µl of anti-testosterone antibody (1:5,000; Covalab, France). Then, the [3H]testosterone tracer (100 µl) was added and samples were incubated at 4°C for another 2 h. Bound and free hormone fractions were separated with dextran-charcoal and the amount of bound testosterone was measured with a scintillation counter. The minimum concentration of testosterone detectable in the medium was 70 pg/ml. The intra- and inter-assay variations, determined as the ratio between the SDs and the mean values of 15 determinations of the same solution containing 1 ng/ml testosterone, were 3% and 10%, respectively.

### Gonadotrophin Immunofluorometric Assay

Plasma was separated and stored at 20°C. Plasma gonadotrophins concentrations were measured by using immunofluorometric assays, as initially described for LH [35] and FSH [36] using 25 µl (LH) and 30 µl (FSH) plasma in duplicate for each sample. To further validate this method we made a dilution series of rat and mouse pituitaries to cover the whole analysis range of the assays. The results show that the samples can be analyzed in a linear manner over the whole standard ranges; 0,04–16,0 ng/ml in the LH assay (rat, R^2^ = 0,9924; mouse, R^2^ = 0,9997) and 1,0–12,5 ng/ml in the FSH assay (rat, R^2^ = 0,9912; mouse, R^2^ = 0,9943). The limits of sensitivity of the assays are 0,03 and 0,1 ng/ml in the plasma for LH and FSH, respectively. The intra assay coefficients of variation are around 10% and 5% respectively for LH and FSH at the concentration levels measured in this study.

### Morphometric analysis of Sertoli, Leydig and germ cells

#### Sertoli cells and gonocytes

**Figure 1:**
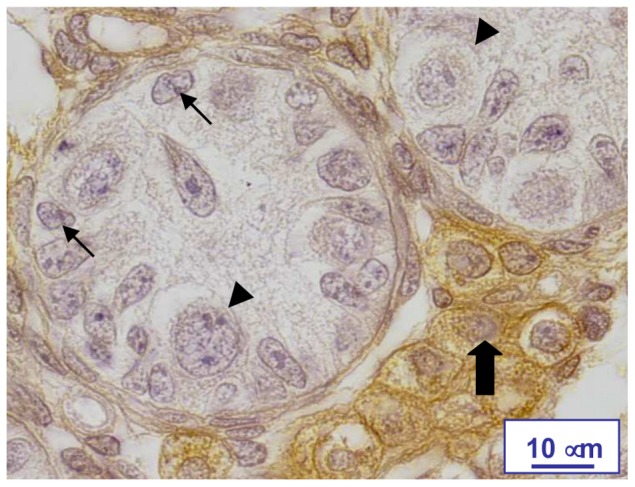
Identification of the testis cell types. Representative microphotograph of a section from a wild type mouse testis at 0 dpp after immunostaining for 3β-HSD and counterstaining with hematoxylin-eosin. The large arrow indicates 3β-HSD-positive Leydig cells. Gonocytes (arrowheads) were identified by their large, spherical, lightly stained nuclei that contain fine chromatin granules and two or more globular nucleoli, and by the clearly visible cytoplasmic membrane. Sertoli cells (smaller arrows) were identified based on their basal position in the seminiferous cords and their cytological features (irregular nucleus and no clear cytoplasmic membrane).

Sertoli cells and gonocytes in testes from FSH-R^−/−^ and control littermates were identified and counted based on previously described morphological criteria (Fig. 1) [12], [37]. The accuracy of the counting method was assessed based on two criteria. First, the minimum number of sections to be counted was identified such that the calculated number of total cells was similar to the one obtained by counting a larger number of sections. Second, we checked that similar numbers of cells were obtained for both testes of the same animal. To this aim, we mounted one out of 20 sections in order to count gonocytes and Sertoli cells in at least nine sections that were equidistantly distributed along the testis. The sum of the values for each testis was multiplied by 20 to obtain the crude count (CC) per testis. The Abercrombie's formula was used to correct for double counting, which results from the presence of a cell in two successive sections: TC = CC×S/(S+D), where TC is the true count, S is the section thickness (5 µm), and D is the true mean diameter of the cell nuclei. D corresponds to the mean of the nuclear diameters measured (DM) on the section divided by π/4 to correct for the over-expression of smaller profiles in sections through spherical particles. DM was calculated in each testis based on at least 100 random determinations with a computerized video micrometer (Microvision Instruments). This counting method has been previously validated [37]. It provides values similar with those obtained by disector methods [29].

#### Leydig cells

Leydig cells were identified by immunohistochemical detection of 3ß-HSD, using an antibody provided by Dr. G. Defaye (INSERM Unité 244, Grenoble, France) (Fig. 1). Immunostaining was performed with the Vectastain Elite ABC kit (Vector Laboratories), as described previously [12], [38]. All 3ß-HSD-positive cells in every 10 sections were counted and the cumulative number per testis thus obtained was multiplied by 10 to obtain the crude count (CC). To correct for any double counting resulting from the appearance of a cell in two successive sections, the Abercrombie's formula was used. This counting method has been previously validated [12], [38]. It provides values similar with those obtained by disector methods [29].

All counts were done blind.

### Measurement of bromodeoxyuridine incorporation

Newborn mice were intraperitoneally injected with 50 mg/kg body weight of a 5-bromo-2′-deoxyuridine (BrdU) and 5-fluoro-2′deoxyuridine solution, according to the manufacturer's recommendations (Cell Proliferation Kit; Amersham, Buckinghamshire, UK) 3 h before sacrifice. BrdU incorporation in proliferating cells was detected by immunohistochemistry, as described previously [33]. The BrdU incorporation index (percentage of labeled Sertoli cells) was obtained by counting at least 1000 Sertoli cells.

### Immunohistochemical analysis of AMH expression

Immunostaining for Anti-Mullerian Hormone (AMH) was performed as previously described using anti-AMH antibodies, kindly provided by N. Di Clemente (INSERM 782, Clamart, France) [37]. To compare the intensity of the staining in FSH-R^−/−^ and controls, FSH-R^−/−^ and FSH-R^+/+^ testis sections were mounted on the same slide and were immunostained in the same drop. Negative controls, without the primary antisera, were included in each experiment and no AMH expression could be detected (not shown).

### RNA extraction and analysis by real-time quantitative PCR (Q-PCR)

Total RNA was extracted from 0, 8 and 10 dpp testes with the RNeasy Micro Kit (Qiagen, Courtaboeuf, France) and reverse transcribed using the QuantiTect Reverse Transcription Kit (Qiagen, Courtaboeuf, France). Q-PCR was then performed on an ABI PRISM 7000 Sequence Detector System using the TaqMan PCR Master Mix (Applied Biosystems, France). AMH, Transferrin, Desert hedgehog (Dhh), Claudin 11 (Osp), P450 Side-Chain-Cleavage (P450scc), Steroidogenic acute regulatory protein (StAR), P450 17α-hydroxylase/c17-20 lyase (P450c17), Gata-6 and ß-Actin transcripts were detected using the following TaqMan gene expression assays (Applied Biosystems, Courtaboeuf, France): Mm00431795-g1, Mm00446708-m1, Mm01310203-m1, Mm00500915-m1, Mm00490735-m1, Mm00441558-m1, Mm00484040-m1, Mm00802636-m1 and Mm00607939-S1. Q-PCR was performed and data analyzed as previously described [33].

As no endogenous control gene specifically expressed in Leydig cells is known, and as the number of Sertoli cells was reduced in FSH-R -/- testes whereas that of Leydig cells was unchanged leading to an increase of the proportion of Leydig cells in the testis, the mRNA expression of steroidogenic genes was normalized to an exogenous internal reference mRNA [20], [39]. Furthermore, this procedure allows standardization for variations in the efficiency of RNA extraction and reverse transcription and for RNA degradation. The reference was 5 ng of luciferase mRNA (Promega) that were added to each testis at the start of the RNA extraction procedure. Briefly, we extracted RNA from each testis into the same volume of buffer (20 µl) and qPCR was performed with the same volume of RNA extracts (0.4 µl). Thus, all RT-qPCR were performed from 2% of the testis and thus from 2% of each cell types contained in the testis. After sample processing, the recovered quantities of luciferase and target mRNAs were independently measured by multiplex real-time PCR and the sample of luciferase mRNA was then used as a normalization factor to control for the loss of mRNA during the cell lysis, RNA isolation, DNA removal steps and reverse transcription. The advantage of this approach is that measurements are expressed per testis i.e. for the same number of Leydig cells.

### Statistical analysis

All values are the mean ± SEM. The significance of the differences between the mean values of body weight was evaluated with the Mann and Whitney U test. Data from FSH-R^−/−^, FSH-R^−/+^ and FSH-R^+/+^ testes were compared using the Student's paired *t*-test or Student's unpaired *t*-test for samples from the same littermate, or different littermates respectively.

## Results

### Morphometric studies

Body weights of the mice were unchanged after FSH-R deficiency at 0 dpp: 1.73±0.08 g in FSH-R^+/+^ (n = 18), 1.83±0.08 g in FSH-R^+/−^ (n = 25) and 1.65±0.07 g in FSH-R^−/−^ mice (n = 29).

### Development and function of Sertoli cells

**Figure 2:**
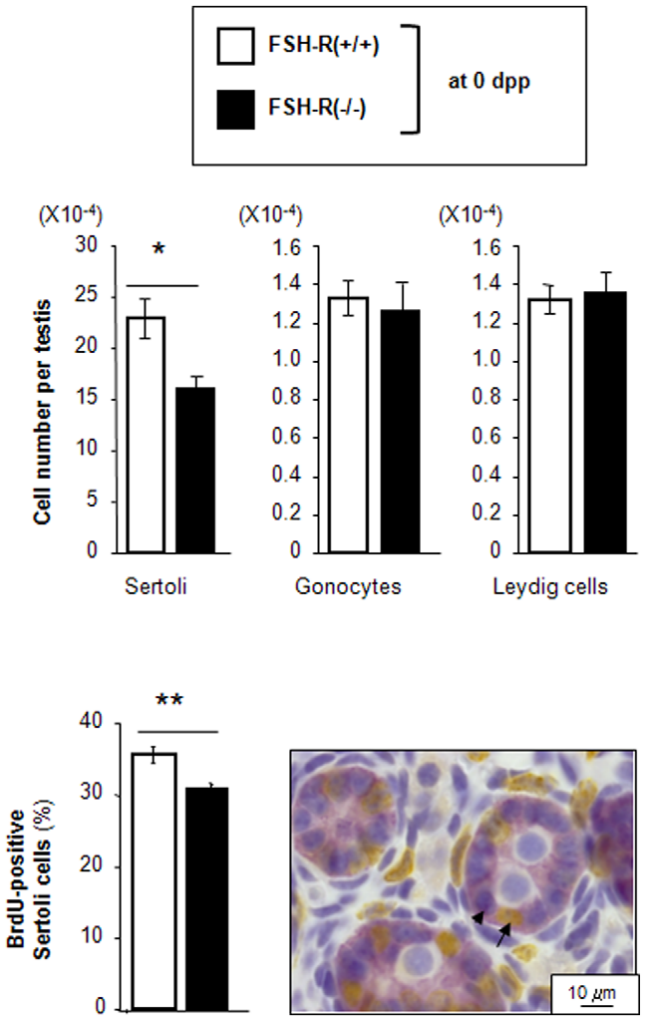
Effect of FSH-R deficiency on the number of testis cells at birth. Upper panel. Number of gonocytes, Leydig cells and Sertoli cells per testis in FSH-R^−/−^ and FSH-R^+/+^ mice at birthday (0 day post partum (dpp)). Values are the mean ± SEM from 5–6 animals for each group. *, *P*<0.05 using the Student's unpaired-*t* test. Lower panel BrdU was injected in 0 dpp mouse neonates 3 h before sacrifice. Testes were fixed and BrdU incorporation in the nuclei of Sertoli cells was determined by immunohistochemistry (right panel). Labeled (arrow) and unlabeled (arrowhead) cells were counted. The identification of Sertoli cells was facilitated by staining with an anti-AMH antibody (pink). The percentage of BrdU-positive Sertoli cells was determined by counting at least 1000 Sertoli cells and presented as mean ± SEM (left panel) ). n = 6 ; **, P<0.01 using the Student's unpaired-*t* test.

At birth (0 dpp), the histology of FSH-R^−/−^ and FSH-R^+/+^ testes was comparable with similar number of gonocytes. Indeed, the total number of gonocytes is already determined by 15.5 dpc, when they enter the quiescent period [3], and thus before the onset of FSH secretion [28]. Conversely, the number of fetal Sertoli cells was reduced by 22% in testes from FSH-R^−/−^ mice in comparison to wild type animals (Fig. 2), whereas the mean diameter of Sertoli cell nuclei was not significantly different. Furthermore, BrdU incorporation in Sertoli cells was significantly lower in FSH-R^−/−^ than in FSH-R^+/+^ testes (Fig. 2).

**Figure 3:**
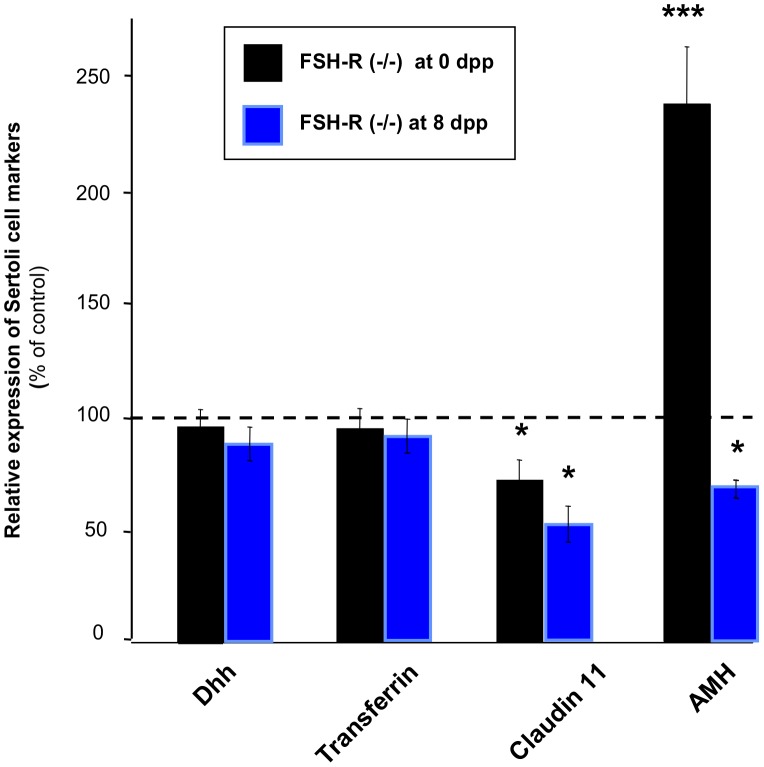
Effect of FSH-R deficiency on the expression of Sertoli cell markers. RNA was extracted from whole testes of FSH-R^−/−^ and wild type mice at 0 and 8 dpp and reverse transcribed. The expression levels of specific Sertoli cell markers were then measured by real time Q-PCR and normalized to *GATA-6* mRNA expression. Results are expressed as the percentage of expression relative to that of wild type controls. Values are the mean ± SEM of 8–9 animals for each group. *, *P*<0.05; ***, *P*<0.001 using the Student's unpaired-*t* test.

**Figure 4:**
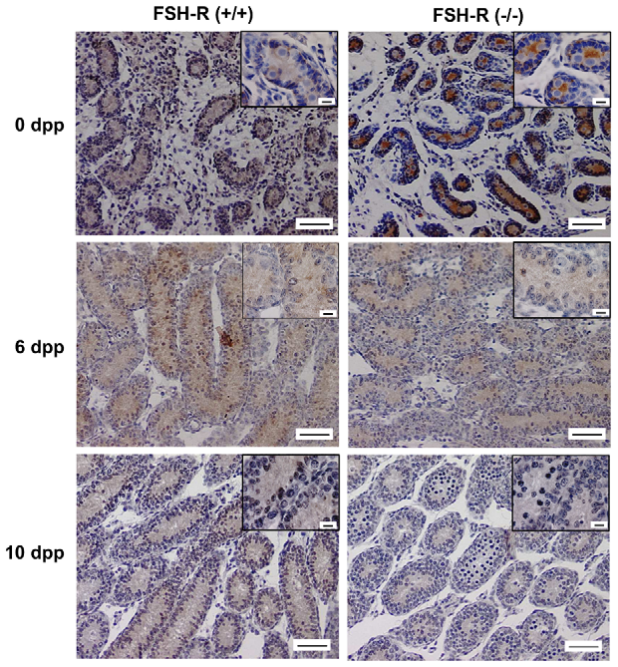
Effect of FSH-R deficiency on AMH expression. Testes were removed from FSH-R^−/−^ and control (FSH-R^+/+^) mice at 0, 6 and 10 dpp, fixed in Bouin's fluid, immunostained for AMH and counter-stained with hematoxylin. Scale bars = 50 µm. *Insets*, higher magnifications : scale bars = 10 µm.

To determine the role(s) of FSH in the function of fetal Sertoli cells, the expression of Sertoli cell-specific markers was assessed by Q-PCR (Fig. 3). The relative expression (normalized to βactin) of the transcription factor *GATA-6*, a member of the GATA-binding protein family that is specifically expressed in Sertoli cells [40], was decreased by 30% in FSH-R^−/−^ neonatal testes in comparison to wild type testes, in line with the 22% reduction in the number of Sertoli cells. This result is in agreement with the fact that *GATA-6* mRNA expression is not FSH-dependent [41]. Therefore, we then normalized the mRNA levels of Sertoli cell marker by using *GATA-6* as an endogenous reference. Both *Transferrin* [42], [43] and *Dhh* mRNA levels were comparable in FSH-R^−/−^ and FSH-R^+/+^ testes throughout post-natal development. Conversely, the expression of *Claudin 11*, which is involved in testis organogenesis and in the development of tight junction intermembranous strands between Sertoli cells [44], was significantly reduced in 0 and 8 dpp FSH-R^−/−^ testes. Finally, AMH was used to determine the differentiation status of Sertoli cells because its expression sharply drops during the perinatal and prepubertal periods [45]. *AMH* mRNA level was 2.3-fold higher in 0 dpp FSH-R^−/−^ testes and was significantly decreased in 8 dpp FSH-R^−/−^ testes in comparison to FSH-R^+/+^ controls. This dynamic change in AMH expression was confirmed by immunohistochemical analysis. In comparison to wild type FSH-R^+/+^ controls of the same age, AMH staining in FSH-R^−/−^ testis sections was clearly stronger at 0 dpp, comparable at 6 dpp (with heterogeneous staining from one seminiferous cord to another) and strongly reduced at 10 dpp in FSH-R^−/−^ testes (Fig. 4).

### Development and function of Leydig cells

**Figure 5:**
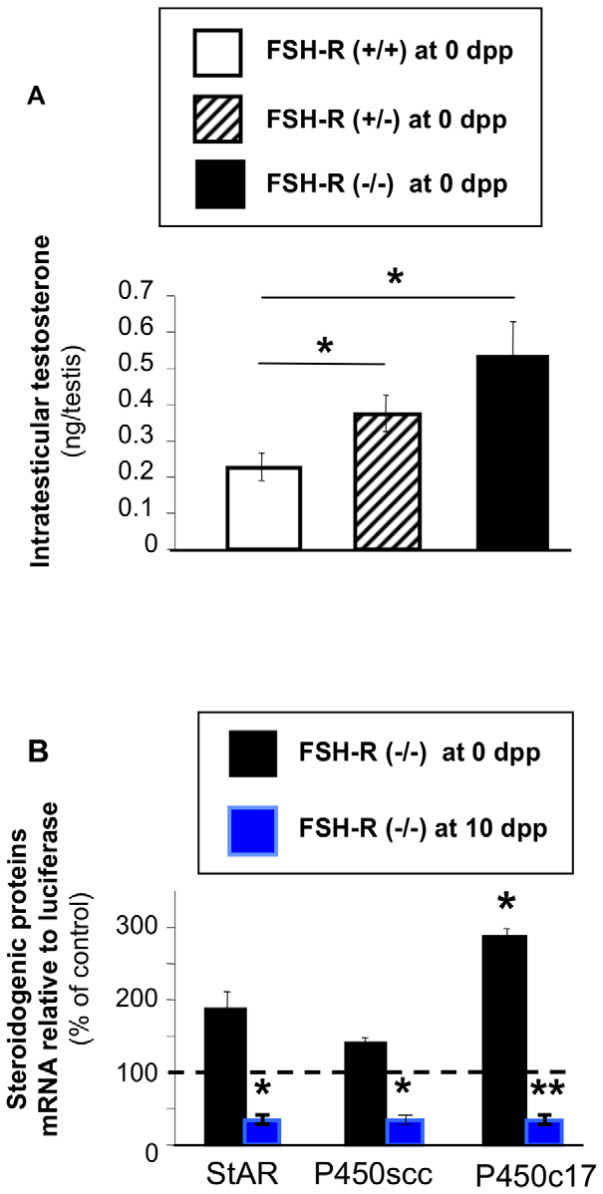
Effect of FSH-R deficiency on testis steroidogenic activity. A: Testosterone concentration was measured in FSH-R^+/+^, FSH-R^+/−^ and FSH-R^−/−^ testes. In 10 litters, one fetus of each genotype was used. Values are the mean ± SEM the 10 animals. *, *P*<0.05 using the Student's paired-*t* test. B: RNA was extracted from whole testes of mice at 0 and 10 dpp and reverse transcribed. The expression levels of specific steroidogenic genes were measured by real time Q-PCR and normalized to an external standard (luciferase). Results are expressed as the percentage of the expression in FSH-R^−/−^ testes relative to that of controls, arbitrarily set at 100%. Values are the mean ± SEM of 5–6 animals for each group. *, *P*<0.05 using the Student's unpaired-*t* test.

At birth, the morphological arrangement of Leydig cells in clusters (Fig. 1) and the number of 3ß-HSD-positive cells was comparable in FSH-R^−/−^ and FSH-R^+/+^ testes of littermates (Fig. 2). Surprisingly, the mean testosterone concentration was significantly higher in testes from FSH-R^−/−^ mice than from wild type littermates, despite the high individual variability (Fig. 5A). To determine the cause of this effect, the expression of genes coding for various proteins involved in testicular steroidogenesis (*P450scc*, *P450c17* and *StAR*) was analyzed by Q-PCR and normalized to an external standard (luciferase). The mean expression level of *P450scc*, *P450c17* and *StAR* at birth was higher in testes from FSH-R^−/−^ animals than from control littermates, and this difference was significant for *P450c17* (Fig. 5B). Conversely, at 10 dpp, when adult Leydig cells appear, their expression was strongly reduced in FSH-R^−/−^ in comparison to wild type testes (Fig. 5B), as previously described [20].

### Determination of LH and FSH plasma levels

**Figure 6:**
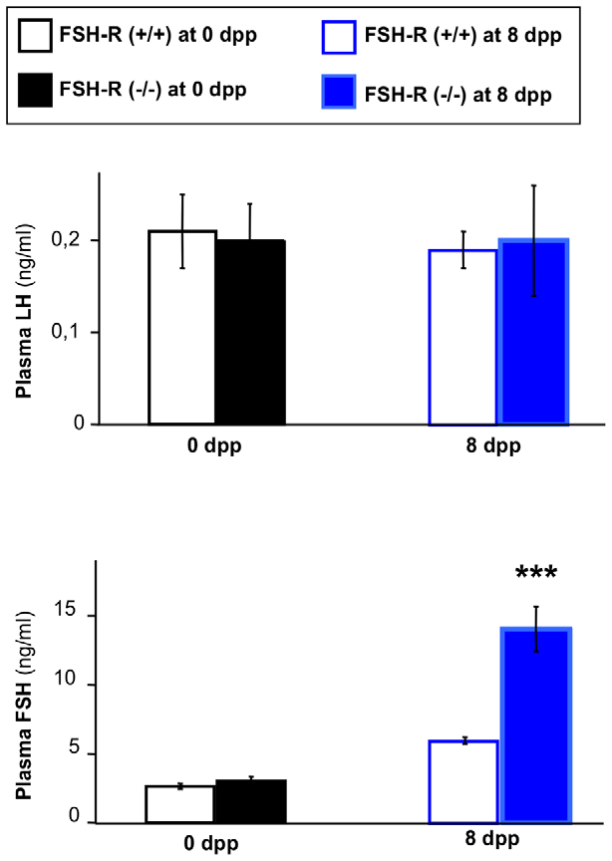
Effect of FSH-R deficiency on LH and FSH plasma levels. LH and FSH concentrations were measured in plasma samples from FSH-R^−/−^ and FSH-R^+/+^ mice at 0 dpp and 8 dpp. Values are the mean ± SEM of 5–6 plasma pools. *, *P*<0.05 using the Student's unpaired-*t* test.

The activity of the hypothalamic-pituitary axis is regulated by a feedback loop operating from the gonads. To determine whether this control was altered in the absence of FSH signaling, we measured the plasma concentration of FSH and LH (Fig. 6). At birth, LH and FSH plasma levels were comparable in FSH-R^−/−^ and wild type animals. Conversely, at 8 dpp, FSH level was increased by 2.3-fold in FSH-R^−/−^ animals, whereas no change in LH levels was detected.

## Discussion

This study shows for the first time that, in the absence of FSH signaling the development and function of Sertoli and Leydig cells are altered during late fetal life in the mouse. Furthermore, many of the effects due to the absence of FSH signaling during fetal life are different or opposite to those observed during prepubertal period.

Sertoli cells divide during the fetal and neonatal periods and cease to proliferate at 17–18 dpp [3], [4]. We found that the number of Sertoli cells and their proliferative index were significantly reduced at birth in FSH-R^−/−^ mice, suggesting that FSH produced during late fetal life promotes the proliferative activity of Sertoli cells. This finding is consistent with the proliferative role of FSH largely previously described during late fetal life in the rat [26], [27]. To evaluate fetal Sertoli cell function and differentiation, we assessed the expression of selected Sertoli cell-specific markers. Claudin 11 is expressed by Sertoli cells and is implied in testicular organogenesis and development of tight junction intermembranous strands between Sertoli cells [44]. The significant reduction of *Claudin 11* mRNA expression in 0 and 8 dpp FSH-R^−/−^ testes is an evidence of the physiological effect of FSH on Sertoli cell function in late fetal life. Conversely, *Transferrin* expression was not affected in mice lacking FSH signaling, indicating that, differently from adult life, FSH is not required for *Transferrin* expression during fetal, neonatal and prepubertal life. Lastly, we tested AMH expression that is a perfect marker for determining the state of Sertoli cell differentiation because its expression sharply drops during the perinatal and prepubertal period [45]. FSH-R deficiency induced a rise in AMH mRNA and protein levels at birth. This is in accordance with our previous work showing that decapitation increases AMH expression during late fetal life in the rat [27]. Furthermore, we show here a clear developmental switch from a negative to a positive action of FSH signaling on AMH expression around post-natal day 6. This finding might help clarifying the contradictory results about the effects of FSH administration on AMH expression described in two previous works. As AMH expression was reported to be reduced at 4 dpp in one study [46] and to be increased at 7 dpp in the other one [45] our finding reconciles these data.

Early differentiation of fetal Leydig cells is independent from pituitary hormones [15], [34]. In the rat, the activity of fetal Leydig cells, but not their differentiation, becomes dependent from LH only during late fetal life [47]. The higher testosterone concentration and *P450c17* mRNA expression in FSH-R^−/−^ than in wild type testes at birth suggest that FSH signaling controls the activity of Leydig cells during late fetal life also in the mouse, although LH plasma levels were comparable in the two groups of mice. The number of Leydig cells was similar in FSH-R^−/−^ and wild type testes at birth. This was expected because the number of Leydig cells does not change significantly between 16 dpc and 5 dpp in mice and FSH is produced only after 17 dpc [28], [29]. Surprisingly, our results show that FSH signaling physiologically inhibits the steroidogenic activity of fetal Leydig cells at birth, whereas it is known to positively regulate the activity of adult Leydig cells [5], [6], [19], [48]. Accordingly, we observed this positive effect of FSH in 10 dpp testes, when adult Leydig cells arise.

As it is generally acknowledged that fetal Leydig cells do not express FSH-R, our results imply that Sertoli cells produce one or more factors that act on Leydig cells during late fetal life. We thus assessed the expression of two important Sertoli cell-derived factors (AMH and Dhh) which may be involved in the regulation of fetal Leydig cell development [49], [50], [51], [52]. Dhh expression was unaffected by FSH-R deficiency, whereas that of AMH was increased. However, the effects of FSH on testosterone production are unlikely to result from an action of AMH because a previous study in the rat demonstrated that AMH has an inhibitory effect on the amount of testosterone produced by fetal Leydig cells [52].

We also investigated the effect of FSH-R deficiency on the plasma gonadotropins levels. We previously observed an increase in LH plasma levels after *in utero* castration of male rat fetuses showing that the fetal testis exerts a negative feedback during late fetal life in the rat [53]. In the present study, no changes could be detected in LH level at both 0 and 8 dpp and in FSH at 0 dpp. This may be due to the weakness of the changes in plasma testosterone and inhibin levels induced by FSH-R deficiency and to the high individual variability in LH plasma level. On the contrary the expected increase in plasma FSH level was observed at 8 dpp.

Our results are different from those described in previous works by O'Shaughnessy's laboratory [20], [30] Specifically, they reported that FSH-R deficiency did not affect Sertoli and Leydig cells number at 1, 5 and 20 dpp and decreased these numbers only in adults. The expression of Claudin 11 was unchanged at 5 and 20 dpp and was decreased only in adults. In the same way, a reduction in AMH expression was observed only after 20 dpp. Similarly, they reported that the testicular concentration of testosterone level was normal in FSH-R^−/−^ animals during neonatal and prepubertal life up to 20 dpp. They concluded that FSH does not control the fetal and early neonatal development and function of Leydig and Sertoli cells. In a very recent paper, O'Shaughnessy and coll. confirmed their previous data [31]. At birth, they observed no significant difference between control and FSH-R KO mice for all the parameters that they measured (testicular volume, tubule diameter, Sertoli, germ and Leydig cells numbers). The number of Sertoli cells was not significantly reduced at 5 dpp and even at 20 dpp. They also studied the effects on testis development of ablating FSH-R in mice with ablation of Androgen Receptors ubiquitously (ARKO) or specifically in the Sertoli cells (SCARKO). Interestingly, they observed that a deletion of FSH-R in SCARKO mice reduced the number of Sertoli cells at birth. But this effect was no longer observed at 5 dpp. Furthermore, deletion of FSH-R in ARKO mice did not significantly change the number of Sertoli cells at birth, at 5 dpp and at 20 dpp. Taken together, this paper suggests that FSH signaling is normally inefficient during fetal and neonatal life but it can work in certain specific conditions.

These important discrepancies with our findings may be due to several reasons. First, the portions of the R-FSH gene deleted are slightly different. O'Shaughnessy *et al.* used FSH-R^−/−^ mice in which exon 1, the 5′ region including the transcriptional start site and part of intron 1 were ablated [54] whereas in our FSH-R^−/−^ mouse model only exon 1 of FSH-R was missing [32]. It cannot be excluded that the deletion of the transcriptional start site would have creates a new transcriptional start site. Second, though both studies used the same mouse strain, ES cells from 129SV mice injected in C57BL/6 blastocysts, recombinations which occurred during backcrosses are different giving rise to different genetic backgrounds. Third, more importantly, O'Shaughnessy *et al.* compared FSH-R^−/−^ animals with FSH-R^+/−^ animals used as controls, whereas our controls were FSH-R^+/+^ mice. Indeed, in accordance with O'Shaughnessy's data, we observed that there is no statistical difference in testicular testosterone content between FSH-R^−/−^ and FSH-R^+/−^ pups, even when using animals issued from the same littermates.

O'Shaughnessy's *et al.* reported some differences in Leydig cell function between FSH^−/−^ and FSH-R^−/−^, suggesting that FSH-R is partly constitutively active [20]. This raises the question of whether the effects we observed are solely the result of FSH-R ablation or can be analyzed as the consequence of the suppression of the action of circulating FSH. Many data argue for the second hypothesis. First, FSH is expressed as soon as late fetal life in the mouse [28]. Second, mouse fetal testes respond to FSH as early as 14.5 dpc in organotypic culture systems [12]. Third, in hypogonadal (hpg) mice, which lack circulating gonadotropins, a reduction in the number of Sertoli cells was observed at day of birth [29]. Lastly, differences between FSH^−/−^ and FSH-R^−/−^ reported by O'Shaughnessy *et al.* never affected any Leydig or Sertoli morphometrical, functional or molecular parameters before 20 dpp suggesting that the constitutive activity of FSH-R does not work during fetal and neonatal life [20], [30].

In conclusion, by invalidating FSH-R in the mouse, we demonstrated for the first time that, at the end of fetal life, FSH signaling and probably FSH itself physiologically regulate the proliferation and differentiation of Sertoli cells and the secretion by Sertoli cells of paracrine factors which modulate Leydig cell functions. Furthermore, most of the FSH signaling-dependent effects observed during fetal/neonatal life differ from those observed in prepubertal testis. This brings a new concept in the endocrinology of the fetal and neonatal testis that is opposite to the usually accepted belief that FSH is not involved in the control of the development of fetal and neonatal testis in the mouse. This reinforces the general concept that the fetal/neonatal testis has its specific endocrine regulations and that it is not a “mini-adult” testis. A better understanding of the endocrinology of fetal testis is now required, especially because it is a very sensitive target of endocrine disruptors [55], [56].
